# Establishment of a novel retinoblastoma (Rb) nude mouse model by intravitreal injection of human Rb Y79 cells – comparison of *in vivo* analysis versus histological follow up

**DOI:** 10.1242/bio.019976

**Published:** 2016-09-30

**Authors:** Alexander V. Tschulakow, Ulrich Schraermeyer, H. Peter Rodemann, Sylvie Julien-Schraermeyer

**Affiliations:** 1 Division of Experimental Vitreoretinal Surgery, Center for Ophthalmology, Eberhard Karls University Tuebingen, Tuebingen 72076, Germany; 2 Division of Radiobiology & Molecular Environmental Research, Department of Radiation Oncology, Eberhard Karls University Tuebingen Tuebingen 72076, Germany

**Keywords:** Retinoblastoma, Xenograft, Mouse model, SLO, OCT, Histology

## Abstract

Retinoblastoma (Rb) is the most frequent primary intraocular tumour in children and, if left untreated, can cause death. Preclinical animal models that mimic molecular, genetic, and cellular features of cancers are essential for studying cancer and searching for promising diagnosis and treatment modalities. There are several models described for Rb, but none of them fully meet our requirements. The aim of this study was to create a novel xenograft-nude mouse-model with broad application possibilities, which closely resembles the clinical observations of Rb patients and which could be used to investigate the development and spread of the tumour by using scanning laser ophthalmoscopy/optical coherence tomography (SLO/OCT) as well as histology methods. We injected human retinoblastoma Y79 cells intravitreally in both eyes of immune-deficient nude mice. The incidences of retinoblastoma as well as growth velocity were analysed 3, 6, 9 and 12 weeks after cell injection *in vivo* by SLO/OCT as well as *ex vivo* by electron microscopy (EM) and hematoxylin/eosin (HE) staining. Moreover, internal organs were histologically screened for potentially occurring metastases. Three weeks post-injection, animals developed a retinoblastoma, and after five weeks tumour growth resulted in swelling of the eyes in individual animals, showing a similar phenotype to that of untreated Rb patients at advanced stages of tumour-development. After 12 weeks, 67.5% of all analysed eyes (29 of 42) contained a retinoblastoma. At early stages of Rb development, the SLO/OCT analysis correlated with the histology results. If the tumours were too large, only histological investigations were feasible. The ultrastructural characteristics of the xenograft-tumours were very similar to those described for patient's tumours. In one mouse, brain metastases were observed. Our retinoblastoma mouse model closely resembles the human disease. SLO/OCT can be used for the detection of Rb at early stages of development and could be used for monitoring the success of future therapies.

## Abstract (precis)

**Summary:** We present a novel retinoblastoma nude xenograft mouse model which closely resembles the human disease and allows broad application possibilities and a comparison of *in vivo* and histological analysis.

## INTRODUCTION

Retinoblastoma (Rb) is the most common primary intraocular malignancy of infancy with an incidence of 1/15,000 to 1/20,000 births. It is estimated that annually Rb affects 7000-8000 new patients worldwide (Broaddus et al., 2009; Seregard et al., 2004), and survival rates vary dramatically. Untreated, mortality is 100%. In 60% of Rb patients, anunilateral Rb tumour is diagnosed at an average age of two years, and in most cases these tumours are not hereditary. In the other 40% of cases, Rb is bilateral and is diagnosed at an average age of one year. All bilateral and multifocal unilateral forms belong to a genetic cancer predisposition syndrome and are hereditary. RB1 gene mutation can be found in all children with a bilateral or familial form, as well as in 10 to 15% of children with a unilateral form of Rb (Jehanne et al., 2014).

Knowledge on tumour genesis was increased enormously with the development of mouse models for retinoblastoma (Villegas et al., 2013). For example, recently, the chemotherapeutic effect of focal melphalan was investigated in the transgenic LHBETATAG murine model. This treatment mediates a significant reduction with respect to the tumour burden, hypoxia and vasculature (Shah et al., 2014).

Ophthalmic imaging, like wide-field photography and echography, are reliable tools, not only in diagnosis but also for detecting regression or progression patterns of Rb (Villegas et al., 2013; Houston et al., 2011; Shields et al., 2009). Intraocular calcifications have also been shown to be analysed successfully using autofluorescence (Ramasubramanian et al., 2011). The role of optical coherence tomography (OCT) was also investigated in the evaluation of fundus tumours in children, and the use of OCT scans during the management of Rb was approved by the clinicians (Houston et al., 2013; Shields et al., 2004; Mallipatna et al., 2015).

The aim of this study was to generate an animal model for Rb that closely resembles the human disease for the purpose of developing new therapeutic options or comparing the efficacy and side-effects of existing treatments. To this aim, retinoblastoma cells of the human Rb−/− cell line Y79 were intravitreally injected into the eyes of immune-deficient nude mice to induce tumour growth. Development and spread of the tumours were characterized by scanning laser ophthalmoscopy (SLO), fluorescein angiography (FA) and OCT, as well as by histology including an analysis at the ultrastructural level, and the *in vivo* and *ex vivo* follow ups were compared. In addition, ultrastructural analysis of the xenograft Rb tumours was performed in order to assess the relevance of our Rb mouse model.

## RESULTS

### Morphological analysis

**Fig. 1.**
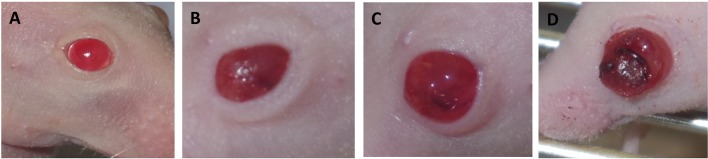
**Results of the morphological analysis.** (A-D) the stages S0-SIII of the mouse eyes: (A) stage 0 (S0): eye of a untreated mouse; (B) stage I (SI): the eye is swollen up to 2× of the normal size; (C) stage II (SII): the eye is swollen up to 3× of the normal size, the eye is cloudy; (D) stage III (SIII): the tumour breaks through the cornea.

Starting at week five after the injection, the eyes began to swell in individual animals. We determined four stages depending on the tumour progression as shown in Fig. 1: Stage 0 (S0) was considered as the morphology of a normal mouse eye. Stage I (SI) was reached after the eye was swollen up to 2× the normal size and showed a cloudy appearance, stage II (SII) was reached after the eye was swollen up to 3× the normal size, stage III (SIII) was reached when the tumour broke through the cornea. Table 1 shows the number of eyes and their corresponding stages at the time points of analysis.

**Table 1.**
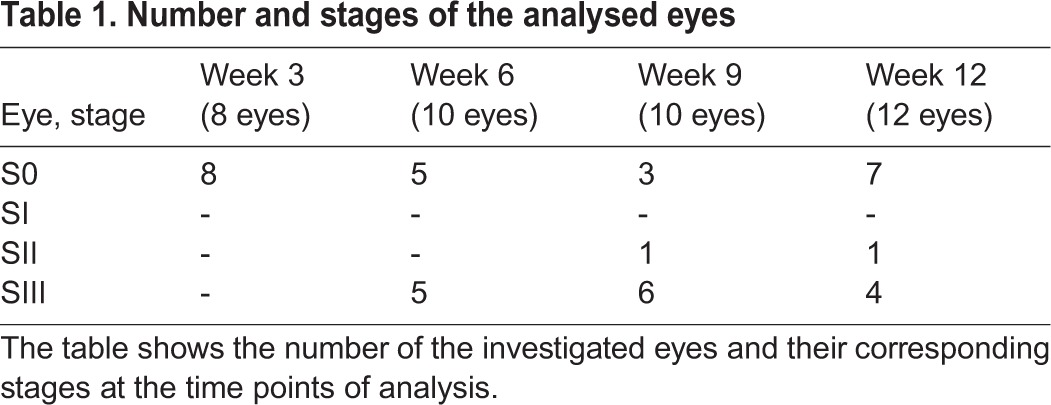
**Number and stages of the analysed eyes**

**Fig. 2.**
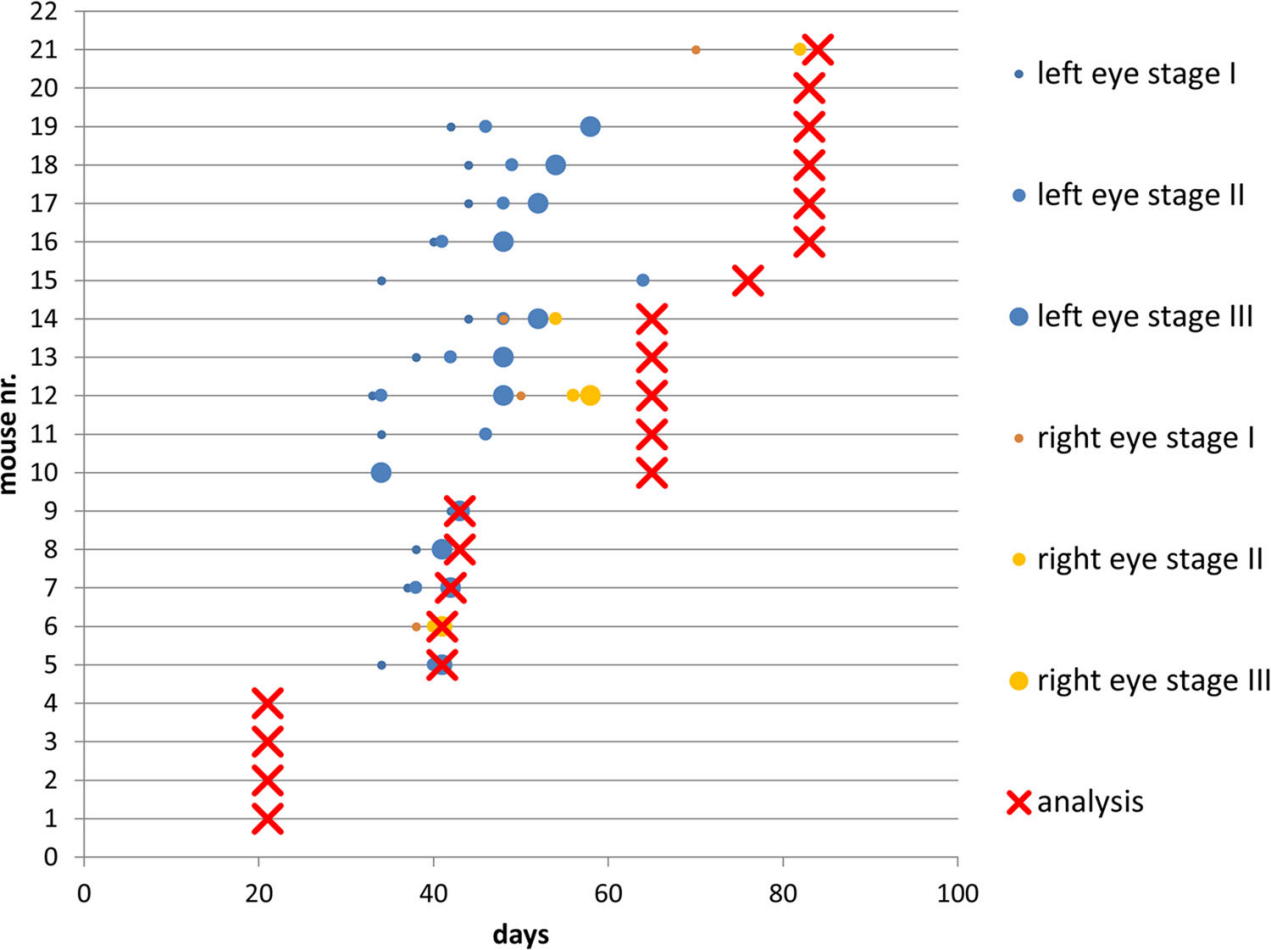
**Overview of the results of the morphological analysis of the mouse eyes during the experiment.** Stages shown are: the beginning of the swelling (small circle=SI); the staging of the eye (medium circle=SII and big circle=SIII); and the time point of analysis (red X) of each eye. Mouse 16 had brain metastasis, the mouse was analysed 35 days after the left eye reached stage III.

As shown in Fig. 2, there were some intra-individual differences concerning the time point of the start and the progress of the swelling of the eyes. The earliest cases of swelling appeared 34 days after injection, and the latest 70 days after injection. In most cases the swelling started between week five and seven after injection and progressed fast from stage I (on average 39 days after injection) to stage II (on average 43 days after injection) and then to stage III (on average 48 days after injection) (Fig. 2).

### *In vivo* imaging using SLO/OCT

The SLO/OCT analysis could only be performed in stage 0 eyes with tumours at very early stages or in eyes without a tumour. In eyes with tumours at later stages of growth (SI-SIII) no analysis was possible, because the tumour covered the fundus.

**Fig. 3.**
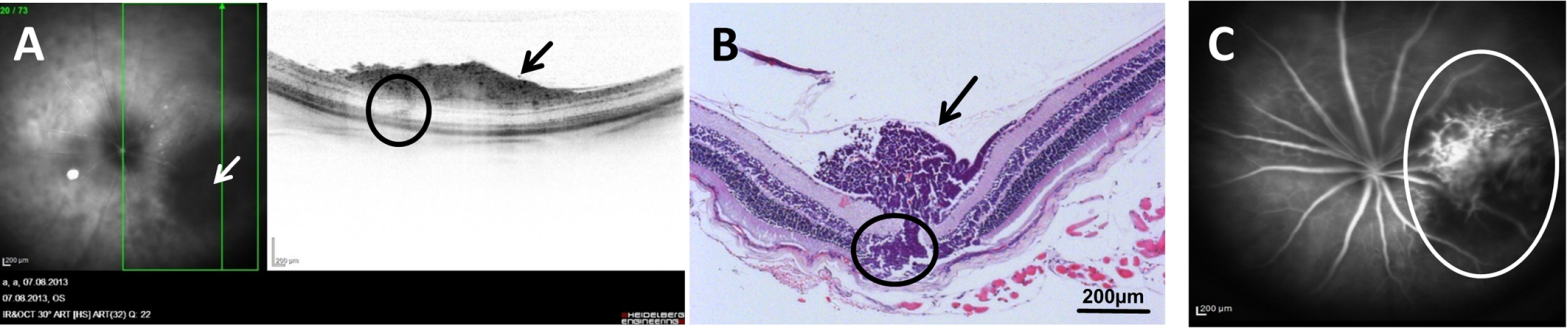
**Results of the *in vivo* and corresponding histological analysis.** Panels (A) and (C) show the results from the *in vivo* analysis of the same tumour-baring eye. The tumour is at an early stage of development, and grew in the vitreous directly on the retina 3 weeks after the injection of the Y79 cells. Left panel in A is the SLO image, the tumour (white arrow) can be seen as a dark region in the lower right corner, the right panel in A shows the OCT image of the green-boxed section in the left panel. (B) The HE-stained sample (×100) of the right panel in A. In both panels A and B, the tumour is shown with black arrows, and located directly on the retina, the area of the tumour breaking through the retina and the area of subretinal tumour growth are circled. (C) SLO image fluorescein mode (FA) 5 min after fluorescein injection (the tumour vessels are circled).

In all cases where SLO/OCT analysis was possible, the results showed a good correlation with the results of the histological analysis. Using OCT, not only could we detect the tumour itself, but could also get information about its growth characteristics. The tumour shown in Fig. 3, for example, broke through the retina and began to grow subretinally, which can be clearly seen on the OCT image (Fig. 3A, left panel) and could later be found on the corresponding HE-stained slide Fig. 3B. The results of the angiography analysis with fluorescein gave a good picture of the tumour's vessel structure Fig. 3C.

### Histological analysis

In the tumour-bearing eyes tumour cells could be observed in the vitreous, retina and subretinal space. An overview of the exact number of eyes and areas of tumour growth up to the time points of analysis is presented in Table 2.

**Table 2.**
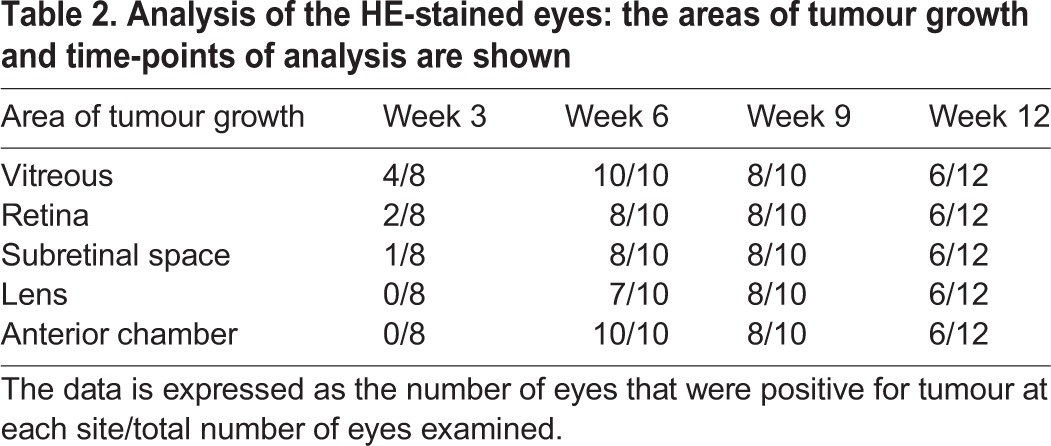
**Analysis of the HE-stained eyes: the areas of tumour growth and time-points of analysis are shown**

For the initial phase of tumour growth (week 3), eight eyes were analysed. Tumour cells could be seen in the vitreous and on the retina in four of them. In two eyes the tumour grew through the retina, in one of these eyes even subretinal tumour growth could be observed.

**Fig. 4.**
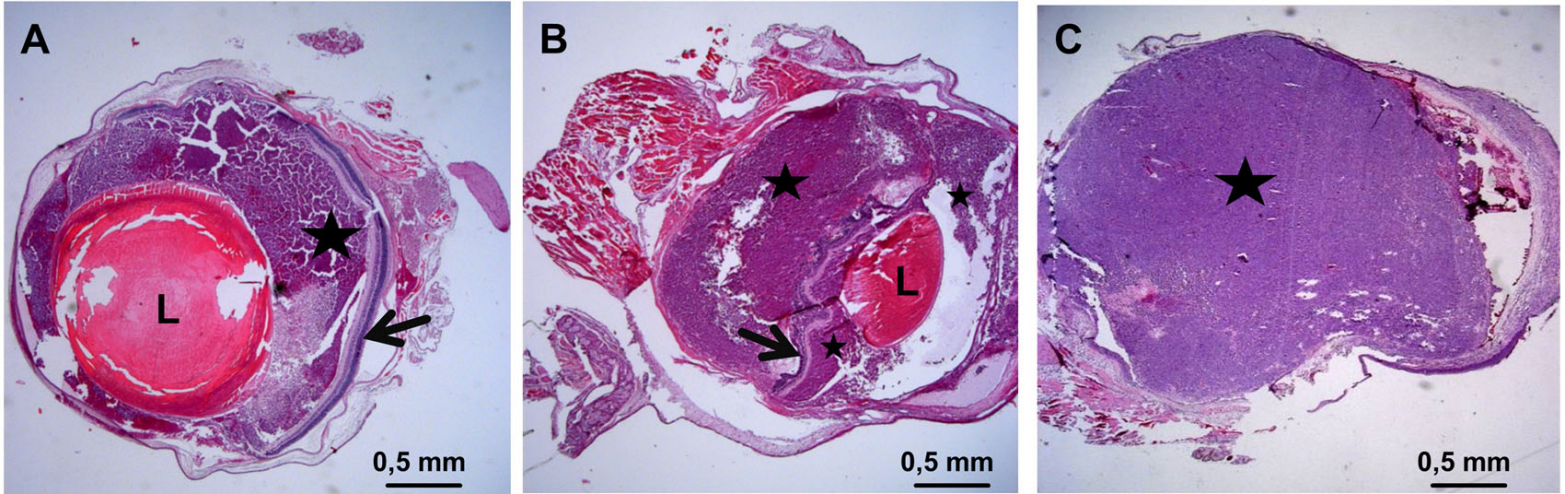
**Results of the histological analysis.** (A) Y79 xenograft tumour from a nude mouse eye 6 weeks after injection (25× magnification), the tumour (black star) grows in the vitreous but does not penetrate the retina (black arrow). (B) Y79 xenograft tumour from a nude mouse eye 6 weeks after injection (25× magnification). Here the tumour, after having grown in the vitreous (smaller black star), penetrated the retina and after strong subretinal growth (bigger black star) pressed the retina (black arrow) in direction of the lens (L). (C) Y79 xenograft tumour from a nude mouse eye 9 weeks after injection (25× magnification), the tumour (black star) has replaced all structures of the inner eye, i.e. the vitreous, the retina and the lens and broke through the cornea (out of sight).

In all tumour-bearing eyes which were analysed 6 weeks after injection, the tumour completely replaced the vitreous and grew into the anterior chamber, in seven eyes the tumour invaded or damaged the lens, and in two eyes the tumour did not penetrate the retina (Fig. 4A). In the other eight tumour-bearing eyes, a subretinal growth could be detected (Fig. 4B).

In advanced tumours (week 9 and 12) the tumour replaced most of the eye's structures, like the vitreous, the lens and retina. Here in all six tumour-bearing eyes the sclera was the only part of the eye's tissue remaining (Fig. 4C).

Histologically, the tumours were composed of typical undifferentiated hyperchromatic cells with scanty cytoplasm having a rosette-like growth pattern, as described for the original tumour (Reid et al., 1974). All tumours showed a high mitotic and necrotic activity.

A tumour was found in 67.5% of the analysed eyes (29 of 42) 12 weeks after the injection of the Y79 cells.

### Metastases

We screened tissues near the tumour, like the brain and skull, for metastases as well as the kidneys, lung, heart, liver, and spleen for the appearance of distant metastases by analysing HE-stained cross sections of these tissues. Only in one mouse could metastases in the brain be found. The metastases were found in the mouse, which after having reached stage III for one eye was kept for the longest period of time (35 days) before being killed and analysed (Fig. 2, mouse 16). In this eye the tumour broke through the sclera in several areas and grew into the brain (not shown). No distant metastases could be found.

### Electron microscopy analysis

**Fig. 5.**
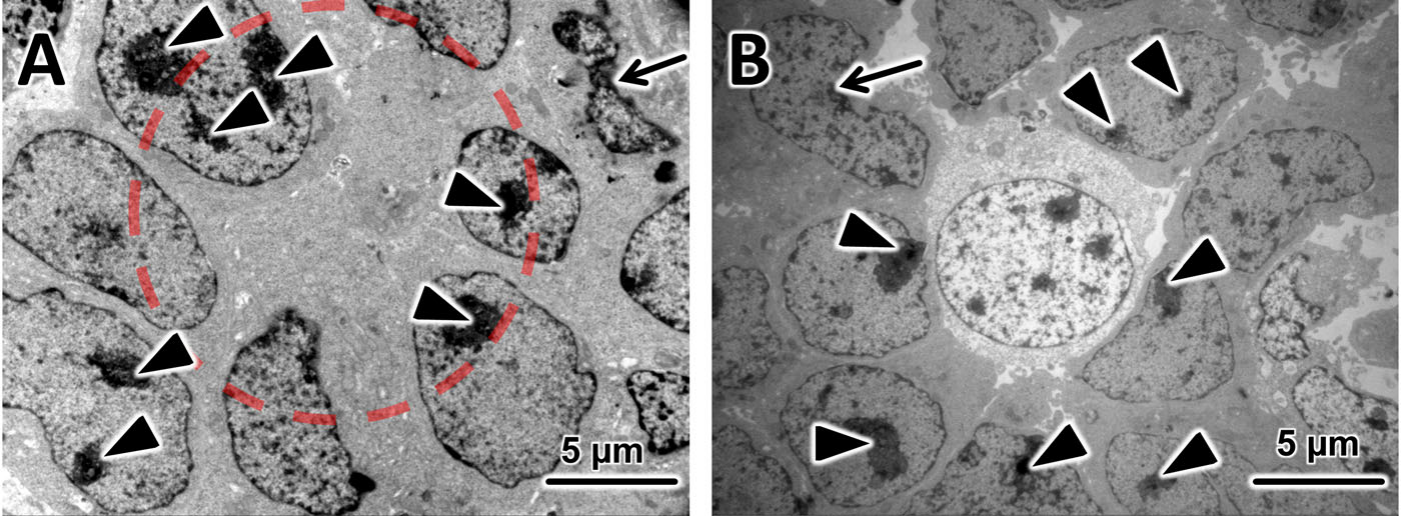
**EM Analysis.** In both electron micrographs the tumor cells show typical large hyperchromatical nuclei with multiple nucleoli (black arrowheads) and elaborate convolutions of the nuclei (black arrows point to cells in which this process is very pronounced). (A) A representative electron micrograph (3000× magnification) from a tissue sample from a xenograft tumour which grew in a nude mouse eye 9 weeks after injection of Y79 cells, here the rosette-like growth of the Y79 cells can be clearly recognized (red circle). (B) A representative electron micrograph (3000× magnification) from the same tissue sample. Here in the centre a necrotic Y79 cell is surrounded by other tumour cells. Black arrowheads point to multiple nuclei; black arrows point to cells with very pronounced elaborate convolutions of the nuclei.

The ultrastructural analysis of the xenograft–tumours, shown in Fig. 5, showed very similar characteristics to those described for the original tumour (Reid et al., 1974), such as poor differentiation but still identifiable rosette-like growth, large hyperchromatical nuclei with multiple nucleoli and elaborate convolutions of the nuclei (Fig. 5A), and numerous degraded and necrotic cells (Fig. 5B) (Reid et al., 1974; Green et al., 1979; McFall et al., 1977). These characteristics are also described as typical for patient's Rb tissues (Rodrigues et al., 1986; Allen et al., 1962).

## DISCUSSION

Preclinical animal models that mimic molecular, genetic, and cellular features of retinoblastoma are essential for studying this type of cancer.

Currently, two types of retinoblastoma animal models exist: transgenic models and xenograft models. The transgenic models have been developed from LH-β-Tag models to conditional gene knock-out models. There are different types of xenograft models, for example orthotopic models and subcutaneous transplantation models. The two types of Rb models present advantages and disadvantages.

The combination of genetic and xenograft models in retinoblastoma research has already help to better understand tumour biology and to find more effective diagnosis and treatments.

Our aim was to create a xenograft mouse model with close resemblance to human Rb tumours which can be used for broad application possibilities including radio therapeutic approaches of Rb treatment.

Literature research indicates that in addition to the use of transgenic animals as a model system for retinoblastoma, another possibility is the use of a xenograft model which is based on the implantation of human retinoblastoma cells into the eye of immunodeficient animals. Indeed, retinoblastoma xenograft models are often created using the cell line Y79. This commercially available human retinoblastoma cell line is derived from a two-and-a-half-year-old patient, who had a maternal history of retinoblastoma.

The implantation can be performed in various compartments of the eye; previously the anterior chamber was often preferred because it is more accessible for both the implantation and subsequent observation (Gallie et al., 1977; Totsuka et al., 1982). However, in patients the tumour starts its growth in the retina and penetrates relatively late into the anterior chamber which is physiologically different to the vitreous body, and where the retinoblastoma first encroaches. Thus, a subretinal injection of the retinoblastoma cells is a better reflection of the situation in humans (del Cerro et al., 1993; Rowe et al., 1992). Unfortunately, this kind of injection can cause damage to the choroid and retina, which can result in an unnatural spread of the tumour.

Another possibility is the intravitreal injection of tumour cells as described by (Chevez-Barrios et al., 2000). After the intravitreal implantation of Y79 cells in Rag2 KO mice, tumours formed in the eye and gradually spread, and later could also be found in the brain. Thus we decided to use an intravitreal injection as well.

The Rag2 KO model developed by Chevez-Barrioz was never used for radiotherapeutic experiments, but the nude mouse model used by Totsuka was (Totsuka and Minoda, 1982), therefore we decided to combine the advantages of both models to develop another model.

After the injection of Y79 cells, tumour cells proliferated first in the vitreous and then formed a clearly localised tumour on and through the retina, not exactly consistent with the retinoblastoma tumours observed in children that originate in the retina; however this particularity is common with the retinoblastoma mouse model developed by Chevez-Barrios et al. (2000). In most cases, the tumour broke through the retina and began to grow subretinally. In two cases the tumour did not penetrate the retina at the 6 week time point of analysis (Fig. 4B).

In contrast to the retinoblastoma mouse model developed by Chevez-Barrios et al. (2000) in which the authors observe metastases resulting from migration of tumour cells up the optic nerve, we observed in our model that the sclera seems to be a strong barrier for the tumour. The tumour needs to grow very large and have a long time to break through the sclera. During our experiment the tumour only penetrated the sclera in one mouse eye and formed brain metastases. The metastases were found in the brain of a mouse, which, after having reached stage III for the left eye, was kept alive for the longest period of time (35 days) before being sacrificed and analysed (Fig. 2, mouse 16 ). However, in the Rag-2 knockout (KO) mice used by Chevez-Barrios et al. (2000), the animals were intravitreally injected with Y79 cells in a similar manner as in our experiment, but the mice already developed metastases 4 weeks after the injection (Chevez-Barrios et al., 2000). These results are consistent with those of other groups, who could show that metastasization metastasesin Rag-2 KO mouse models are stronger than in nude mice for several human cancer xenografts like sarcoma (Nanni et al., 2010), breast cancer (Nanni et al., 2012) or adenocarcinoma (Ye et al., 2015). This should be considered when choosing a model. Despite the mentioned differences of the metastasization process in Rag2 KO and nude mice, Gallie et al., described a metastasization of the optic nerve and brain in cyclophosphamide pre-treated nude mice (Gallie et al., 1977); unfortunately the authors do not make any statement about the time point of analysis. In our experiment we had to kill the animals at the latest 12 weeks after tumour cell injection due to the ethical requirements of local authorities, and we consider it very likely that they might develop metastases at a later time point.

A very important aspect of this work was the use of *in vivo* approaches like SLO/OCT for the detection and characterisation of tumours in the mouse eyes and the comparison of the results with the corresponding results of the histological analysis, which showed a good correlation as shown in Fig. 3. A similar funduscopy/OCT-based approach was used for the analysis of the tumours in the eyes of a TAg-RB mouse model by Wenzel et al. with similar results (Wenzel et al., 2015).

In ophthalmological research, *in vivo* analysis like SLO/OCT allows multiple analysis of dynamic biological processes like tumourigenesis, tumour growth and angiogenesis at certain time points in individual animals and can help to reduce the number of experimental animals used.

In conclusion, we showed that our Rb mouse model mimics the human disease. The xenograft tumour samples from our model showed very similar growth characteristics, cellular appearance and ultrastructural characteristics to those described for Rb patient tumour tissue samples. This makes our model a promising tool for the study of retinoblastoma and its potential therapy approaches.

We also show that SLO/OCT can be used for the detection of tumours at early stages of development and could be used for monitoring the future therapies.

## MATERIALS AND METHODS

### Cell culture

The Y79 retinoblastoma cell line originates from a primary tumour of a two-and-a-half-year-old Caucasian female with a maternal history of retinoblastoma in 1971 (Reid et al., 1974).

The human retinoblastoma Y79 cell line was purchased at American Type Culture Collection (ATCC, USA). The cells were cultured in RPMI-1640 medium (Gibco^®^, Darmstadt, Germany) supplemented with 10% fetal bovine serum and 2 mM L-glutamine. The cells grew as a suspension culture and were cultured and passaged as recommended by the ATCC. For the injection, cells from passage 4 were used.

### Intravitreal injection

24 BALB/c nude mice [female, 3 months old, purchased at Janvier (Laval, France)] were used for the study. The animals were kept in individually ventilated cages (IVC) in our animal facility.

The mice were handled at all times in accordance with the German Animal Welfare Act and were under the control of the Animal Protection Agency and under supervision of veterinarians of the University of Tuebingen. The experiments were approved by the local authorities (Regierungspräsidium Tuebingen AK 6/12).

Each animal was first anaesthetized with an intraperitoneal injection of a three component narcosis (0.05 mg fentanyl, 5.00 mg midazolam and 0.5 mg of medetomidine/1 kg body weight, prepared by the Animal Protection Agency of the University of Tuebingen).

The pupils were dilated with 1 to 2 drops of Medriaticum drops (Pharmacy of the University of Tuebingen, Germany) and a drop of topical anesthetic Novesine (OmniVision, Puchheim, Germany) was applied. Methocel (OmniVision, Puchheim, Germany) eye drops were used to avoid drying of the eyes. Injections were performed using a surgical microscope. Two microlitres of sterile phosphate buffered saline (Gibco^®^, Darmstadt, Germany) containing 2×10^4^ Y79 human retinoblastoma cells were injected into the vitreous of each eye through the sclera using a Hamilton syringe with a 26 gauge cannula. Special care was taken to prevent lens damage or posterior retinal punctures. After the injection, the eyes were treated with antibiotic eye drops (Gentamicin-POS^®^, Ursapharm, Saarbrücken, Germany). Finally the mice were subcutaneously injected with an antidote (1.2 mg naloxon, 0.5 mg flumazenil, 2.5 mg atipamezol/1 kg body weight, prepared by the Animal Protection Agency of the University of Tuebingen) which neutralized the anaesthetic.

The animals were examined 2, 12 and 24 h after surgery and then daily. Clinical findings regarding the presence of tumour were recorded.

### *In vivo* imaging using SLO/OCT

Three, six, nine and twelve weeks after injection, groups of five mice were formed. Mice which showed tumour-caused phenotypical changes were primarily analysed. For the analysis a Spectralis™ HRA+OCT SLO/OCT device was used (Heidelberg Engineering, Heidelberg, Germany). The whole procedure was performed as described in Huber et al. (2009) and Fischer et al. (2009). Briefly, the Spectralis^®^ was remodelled to make it usable for the analysis of small rodents by fixing a 78 dpt double aspheric lens (Volk Optical, Inc., Mentor, OH 44060, USA) directly to the outlet of the device, and an additional custom-made 100 dpt contact lens directly on the eyes of the mice. The mice were anaesthetized by a peritoneal injection of a three component narcosis (as described above), and the pupils were dilated with 1 to 2 drops of Medriaticum (Pharmacy of the University of Tuebingen, Germany). Methocel (OmniVision, Puchheim, Germany) eye drops were used to avoid drying of the eyes and to ensure the adherence of 100 dpt- lenses on the mice eyes. The mice were put in front of the device on the XYZ-table and positioned for the analysis. The mice were covered with cloth to avoid hypothermia.

After positioning, the SLO images were taken. In cases when a tumour was detected, an angiography analysis was also performed. 25 µl of a 2% solution of Fluorescein^®^ 10% (Alcon Freiburg, Germany) was given subcutaneously to the mice to make it possible to visualize the retinal and tumour vessels using the FA (fluorescein angiography) mode of the SLO device. After that the OCT-imaging was performed. A detailed protocol for anaesthesia and imaging is described elsewhere (Huber et al., 2009).

### Histological analysis

Directly after the *in vivo* analysis the mice were killed by cervical dislocation. One eye, the brain, lungs, heart, kidney, spleen, and liver of each mouse were immediately fixed in 4.5% formalin containing fixation solution (4.5% Roti Histofix, Carl Roth, Karlsruhe, Germany). The tissues were processed and embedded in paraffin using conventional automated systems. The blocks were cut to obtain serial 4 µm thick sections and stained with conventional hematoxylin-eosin (HE). The slides were examined by the means of a light microscope.

### Light and electron microscopy (EM)

The other eye of each mouse was fixed in 5% glutaraldehyde for electron microscopic analysis. After the fixation (min. 3 days) the eyes were screened under a binocular for areas of interest (aoi), especially tumour-containing areas. The samples containing these aoi were cut (1 mm×1 mm). These specimens were post-fixed with 1% OsO4 at room temperature in 0.1 M cacodylate buffer (pH 7.4), *en bloc* stained with uranyl acetate and lead citrate, and embedded in Epon after dehydration in a graded series of acetones. Semi-thin sections (0.2 µm) were stained with Toluidine Blue and examined by light microscopy (Zeiss Axioplan2 imaging, Zeiss, Jena, Germany). For electron microscopy, the sections were cut ultrathin (0.07 µm) and analysed with a Zeiss 902 A electron microscope (Zeiss, Jena, Germany).
